# Targeting Lipooligosaccharide (LOS) for a Gonococcal Vaccine

**DOI:** 10.3389/fimmu.2019.00321

**Published:** 2019-02-27

**Authors:** Sunita Gulati, Jutamas Shaughnessy, Sanjay Ram, Peter A. Rice

**Affiliations:** Division of Infectious Diseases and Immunology, Department of Medicine, University of Massachusetts Medical School, Worcester, MA, United States

**Keywords:** *N. gonorrhoeae*, Lipooligosaccharide, vaccine, peptide mimic, complement

## Abstract

The increasing incidence of gonorrhea worldwide and the global spread of multidrug-resistant strains of *Neisseria gonorrhoeae*, constitute a public health emergency. With dwindling antibiotic treatment options, there is an urgent need to develop safe and effective vaccines. Gonococcal lipooligosaccharides (LOSs) are potential vaccine candidates because they are densely represented on the bacterial surface and are readily accessible as targets of adaptive immunity. Less well-understood is whether LOSs evoke protective immune responses. Although gonococcal LOS-derived oligosaccharides (OSs) are major immune targets, often they undergo phase variation, a feature that seemingly makes LOS less desirable as a vaccine candidate. However, the identification of a gonococcal LOS-derived OS epitope, called 2C7, that is: (i) a broadly expressed gonococcal antigenic target in human infection; (ii) a virulence determinant, that is maintained by the gonococcus and (iii) a critical requirement for gonococcal colonization in the experimental setting, circumvents its limitation as a potential vaccine candidate imposed by phase variation. Difficulties in purifying structurally intact OSs from LOSs led to “conversion” of the 2C7 epitope into a peptide mimic that elicited cross-reactive IgG anti-OS antibodies that also possess complement-dependent bactericidal activity against gonococci. Mice immunized with the 2C7 peptide mimic clear vaginal colonization more rapidly and reduce gonococcal burdens. 2C7 vaccine satisfies criteria that are desirable in a gonococcal vaccine candidate: broad representation of the antigenic target, service as a virulence determinant that is also critical for organism survival *in vivo* and elicitation of broadly cross-reactive IgG bactericidal antibodies when used as an immunogen.

## Introduction

Gonococcal vaccine development is challenging because the correlates of immune protection are not fully known (1); mechanisms of protective immunity against gonococcal infection in humans are also unknown. Potential mechanisms focus on: (1) antibody (Ab) binding to *Neisseria gonorrhoeae* (*Ng*) together with complement activation that results in direct killing (bactericidal activity) of the organism (2, 3); (2) Ab binding and complement activation to enable opsonophagocytic killing (2); (3) Ab binding to prevent adhesion or invasion (4) and (4) T cell help. Beneficial T_H_1 responses predominate in several successful vaccine approaches that use a female mouse model of gonococcal infection (5–7).

Gonococcal surface molecules that may be appropriate vaccine targets often are antigenically variable and modify epitopes by antigenic or phase variation (8), which complicates vaccine development by creating an ever-changing bacterial surface. The ability to modify surface determinants is beneficial for gonococci and results in evasion strategies to increase fitness and facilitate adaptation of organisms to their environment. Unfortunately, in human infection, adaptive immune responses directed against conserved antigens fail to elicit protection against future bouts of infection; in fact, repeat infections are common, not only because of re-exposure to unidentified infected partners (9) but also because robust protective immune responses are not elicited. Immune responses that do occur may contain subversive elements that enhance the risk for future infection (10, 11). In female mice, experimental gonococcal infection can suppress the development of adaptive immune responses by inducing regulatory cytokines TGF-β and IL-10 and type 1 regulatory T (Treg) cells (12). Intravaginal treatment of infected mice with IL-12 induces persistent immunity against gonococcal reinfection, which is dependent on the production of IFN-γ and antibodies (13) that results in an enhanced T_H_1 response, accelerates clearance of infection and elicits a memory response that results in protection (14).

A number of gonococcal surface components that elicit bactericidal antibodies are under examination as vaccine candidates [reviewed and tabulated (15)]. Immunization with gonococcal outer membrane elicits diverse vaginal and serum antibodies, which can be bactericidal and accelerate clearance of experimental infection (16); however, this approach is not always reproducible (5). An alternative successful approach that favored a T_H_1 response, employed mice immunized (primed) with PorB (the gonococcal major outer membrane protein)-expressing Venezuelan equine encephalitis (VEE) virus replicon particles (VRPs), followed by boosting with recombinant Por B (rrPorB) (5, 17). However, elicited antibodies were non-bactericidal.

Several promising vaccine candidates do not elicit bactericidal antibody activity in natural infection but were predicted to be potential vaccine candidates because a more robust immune response may be forced by vaccination that does not occur in natural infection. In addition, bactericidal antibody responses to several antigens may target important physiologic functions that, if disrupted, could compromise *N. gonorrhoeae* further, including colonization and invasion (4, 18–27), nutrient acquisition (28–35), and immune evasion (36–42). Vaccine candidates that elicit bactericidal antibodies have also been identified by proteomic analysis of *N. gonorrhoeae* surface proteins (43) and, for example, by bioinformatic analysis, in *N. gonorrhoeae*, of an adhesin complex protein (ACP) homolog, originally identified in *N. meningitidis* (4). Other vaccine candidates that target function but are not known to elicit bactericidal activity are also discussed in two reviews (44, 45). A recent study surrounding the epidemic of group B *N. meningitidis* infection in New Zealand calculated cross-protective efficacy of 31% against gonorrhea in persons, aged 15–30, who were administered a Group B meningococcal outer membrane vesicle (OMV) vaccine (46), which subsequently has formed the basis of a licensed Group B meningococcal vaccine. Human vaccination with the licensed vaccine elicits antibodies against *N. gonorrhoeae* (47) but they are non-bactericidal (48).

A successful vaccine candidate(s) may exhibit: i) a broadly representative antigenic target(s); ii) a virulence determinant(s) (for example a determinant(s) that facilitates host evasion) that can be neutralized and iii) a determinant(s) that is critical for gonococcal survival. Such a “triple threat” candidate may prove to be a useful strategy to “corner” a skillful organism that employs numerous mechanisms to escape selective pressure. Successful single antigens used as vaccines against bacteria are the capsular polysaccharides (49–52). While these are not present in *N. gonorrhoeae*, saccharide determinants are present in gonococcal lipooligosaccharides (LOSs).

## Lipooligosaccharide (LOS) Structure

**Figure 1:**
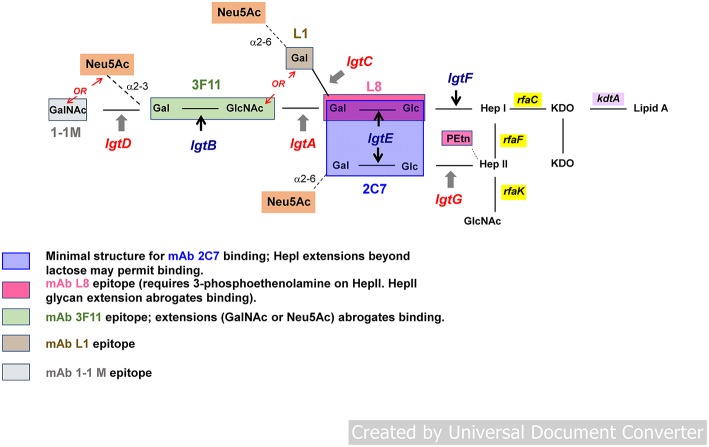
General structure of gonococcal lipooligosaccharide (LOS). Gonococcal LOS consists of three oligosaccharide (OS) chains. The OS chains branch from two heptose residues attached to lipid A via two 2-keto-3-deoxy-mannooctulosonic acid (KDO) molecules. One OS chain elongates from the first heptose (Hep I) outward; two chains extend from the second heptose (Hep II). Lacto-*N*-neotetraose structure (Galβ1-4GlcNAcβ1-3Galβ1-4Glcβ1-4-) or P^k^ (Galα1-4Galβ1-4Glcβ1-4-) extend outward from Hep I. Phase variable genes involved in LOS biosynthesis (*lgtA, C, D, and G*) are shown in red; non-variable genes (*lgtF, lgtE, and B*) in blue. Neu5Ac (sialic acid) is shown in the orange boxes. Sialylation of LOS occurs via α2-6 or α2-3 linkage to galactose (Gal) residues. LOS branching is terminated (“capped”) either by Neu5Ac (sialic acid) or otherwise extend(s) outward by adding hexose(s). LOS epitopes are defined by mAbs 2C7, L8, 3F11, L1, and 1-1M.

Gonococcal LOSs consist of three oligosaccharide (OS) chains, attached to a lipid A core. The OS chains branch from two heptose residues attached to lipid A via two 2-keto-3-deoxy-mannooctulosonic acid (KDO) molecules. One OS chain elongates from the first heptose (Hep I); the 2nd and 3rd chains are connected to the second heptose (Hep II) (Figure 1). The number of branches and the length of OSs in each branch vary among gonococcal strains and, indeed, in the same strain during growth *in vitro* and *in vivo*. The *rfaC* gene that encodes heptosyl transferase is required for the addition of Hep I to KDO (53) (*rfa* genes encoding heptosyl transferases are blocked in yellow in Figure 1).

The *rfaF* gene product, also a (different) heptosyl transferase, adds Hep II to Hep I and is required for elongation (schematically depicted as outward in Figure 1) of the Hep I chain (54, 55). The synthesis of OS chains is modulated by a series of LOS glycosyl transferases (products of *lgt* genes). *lgtF, lgtE, lgtA, lgtB*, and *lgtD* genes are required for stepwise addition of each hexose [or hexosamine in the case of *lgtA* and *lgtD* (shown in Figure 1)] to extend the Hep I chain (Figure 1) (56, 57). In place of full extension, the *lgtC* gene encodes α-galactosyl transferase that terminates Hep l with galactose (Gal), that can undergo sialylation (shown as Neu5Ac in the orange boxes in Figure 1), creating a shorter chain (Galα1-4Galβ1-4Glcβ1-4-) attached to -Hep I (also called the P^*K*^-like LOS) (58). Expression of distinct LOS structures on the gonococcal surface is controlled by the phase variable expression of the LOS glycosyltransferases genes *lgtG, lgtA, lgtC*, and *lgtD* (54, 59) (indicated in red in Figure 1). These genes (*lgtA, lgtC*, and *lgtD*) contain homopolymeric tracts of guanine poly (G), and in the *lgtG* gene, a cytosine poly (C) tract (56, 59–62). Slipped strand mispairing during DNA replication can result in alteration in coding sequences, which leads to premature termination of the corresponding genes and loss of function of the encoded glycosyl transferase proteins resulting in truncated LOS structures. Phase variation of LOS results in changes in size of the predominant LOS structures that are expressed both *in vitro* and *in vivo*. LOS undergoes phase variation at a frequency of 10^−2^-10^−3^ when gonococci are grown in culture (63, 64). Identification of several of these individual structures on the surface of *Ng* can be demonstrated by reactivity with LOS-specific mouse monoclonal antibodies (mAbs) (depicted by colored boxes in Figure 1 and in the legend). Several of the antigenic determinants share structure with human glycosphingolipids (GSLs) (58, 65, 66). The lacto-*N*-neotetraose structure (four sugars extending from -Hep I: [Galβ1-4GlclNAcb1-3Galβ1-4Glcβ1-4-]) recognized by mAb 3F11, is identical to human erythrocyte GSLs (67–69). The alternative Hep I structure, digalactoside (Galα1-4Galβ1-4Glcβ1-4-, the P^K^ structure or the L1 meningococcal serotype (Figure 1), is recognized by mAb L1 and is similar in structure to human paraglobosides (58). The fully extended Hep I, a pentasaccharide (GalNAcβ1-3Galβ1-4GlclNAcβ1-3Galβ1-4Glcβ1-4-), defined by mAb 1-1-M (70, 71), has a structure identical to human asialo-G3 ganglioside (Figure 1) (69). This mimicry may enable *Ng* to avoid immune recognition; antigenic determinants that share structure with human GSLs, therefore, may not be suitable to elicit a response that is specific for the organism, nor would a response to shared human antigens be desirable.

Nevertheless, *Ng* LOSs possess two epitopes, which do not cross react with human GSL antigens. The first comprises Galβ1-4Glc (lactose), the first two hexoses that are β-linked to Hep I (72–74). Together with a phosphoethanolamine (PEtn) substitution at the 3- (cyclic) position on Hep II (as occurs when *lgtG* is OFF and Hep II is not substituted at the 3-position with glucose [Glc]), this structure is recognized by mAb L8 (75). The second epitope is a composite of the first epitope (L8) plus a Galβ1-4Glc (also lactose) that is α-linked to Hep II (area shaded in blue in Figure 1) and represents the minimal structure [*N*-linked fatty acids in lipid A are required for maximal expression (66)] of the epitope recognized by mAb 2C7 (66, 76), called the 2C7 epitope. Absence of Hep II linked lactose (and therefore the complete 2C7 structure/epitope) severely attenuates gonococcal infection in the mouse cervico/vaginal colonization model (7, 77, 78). 2C7 expression therefore, may be an important virulence factor that enhances or may be required for survival and productive infection in humans. Despite phase variation of the *lgtG* gene, that initiates production of the 2C7 epitope (expression is completed by *lgtE*, which is constitutively expressed and adds Gal to Hep II linked Glc) the epitope is widely shared and expressed by most gonococci including 95% of minimally passaged *Ng* clinical isolates (Boston) (2) and in 100% of isolates in Nanjing, China (78). In Nanjing, female subjects who were exposed and infected with *Ng* developed significantly higher levels of 2C7 Ab compared with control women who possessed minimal or no measurable 2C7 Ab. Furthermore, as expected, there was no difference in 3F11 (a self-antigen) antibody levels in infected women vs. controls; neither were there differences in L8 antibody levels between the two groups, all of whom possessed either minimal or no measurable antibody to 3F11 and L8 epitopes. The 2C7 epitope, therefore, is immunogenic in natural infection, more so than at least two other LOS structures that have been antigenically defined.

## LOS Sialylation and Complement Resistance

**Figure 2:**
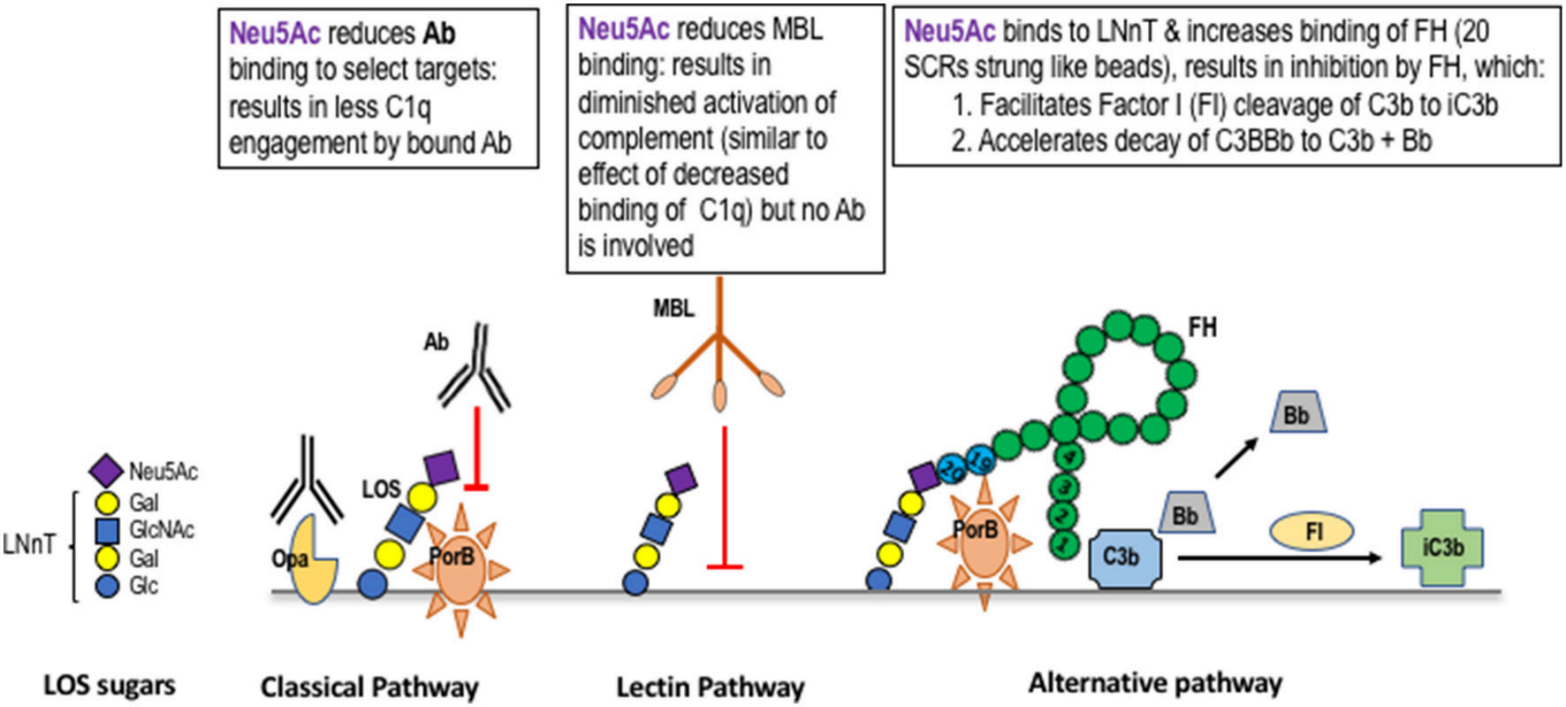
Mechanisms of complement regulation by sialic acid on gonococcal lacto-*N*-neotetraose (LNnT). Gonococci can add N-acetylneuraminic acid (Neu5Ac; the form of sialic acid found in humans) to the terminal Gal of the lacto-*N*-neotetraose (LNnT) LOS structure (a schematic of sialylated LNnT is shown on the left side of the Figure). The presence of Neu5Ac on LNnT LOS reduces the binding of IgG to select targets. As an example, binding of mAbs to PorB, but not to Opacity protein (Opa), is inhibited (79). Sialylation of LNnT also inhibits binding of “natural” IgG in NHS to the gonococcal surface (80). Binding of mannan binding lectin (MBL) to the surface of gonococci is inhibited by LNnT LOS sialylation (81). MBL binds to gonococcal LOS that terminates in GlcNAc (82), which is elongated (“capped”) by Gal and Neu5Ac (shown on the left side of the Figure) and to Opa and PorB (83) [Opa and PorB (shown in the Classical Pathway frame to the left of the Lectin Pathway frame in the Figure)]. Neu5Ac that caps LNnT also regulates the alternative pathway of complement by enhancing binding of factor H (FH; shown as a “string of beads” in the Alternative Pathway frame) (40). Enhanced FH binding to sialylated gonococci is restricted to the LNnT structure; sialylation of the P^k^-like LOS (84), or lactose on HepII (78) does not enhance FH binding. Binding of FH is also dependent on expression of PorB (85) and occurs through the C-terminal domains of FH (SCR18-20) (86). Bound FH acts as a cofactor in the factor I (FI) cleavage of C3b to iC3b (cofactor activity) and also irreversibly dissociates the C3 convertase, C3bBb (decay accelerating activity).

Gonococci “cap” LOS molecules in which Hep I terminates with the lacto-*N*-neotetraose structure (four sugars extending outward from Hep I [the LNnT structure]; Figure 1). Sialylation can occur using the organism's own endogenous sialyltransferase and appropriate sialic acid substrate(s) present in the mammalian genital tract. *In vitro* (exogenous) cytidine monophospho-*N*-acetylneuraminic acid (CMP-Neu5Ac) serves as a suitable substrate. Sialylated gonococci are endowed with several means to enhance pathogenicity. Sialylation of gonococcal LOS inhibits all three pathways of complement through several independent mechanisms: the classical pathway is inhibited by reducing antibody binding and possibly by reducing C1q (the first component of complement) engagement by bound antibody; the lectin pathway is inhibited by reducing mannose binding lectin (MBL) binding; the alternative pathway is inhibited by increased binding of FH, a major soluble down-regulator in the alternative pathway (Figure 2). Sialylation of gonococcal LOS also decreases opsonic killing of gonococci (87–89) in part, because of decreased complement activation and C3 fragment deposition on the surface of sialylated bacteria (38, 40). Sialylation of gonococcal LOS markedly reduces opacity-associated protein (Opa)-mediated invasion of *N. gonorrhoeae* into human epithelial cell lines (90–92). Finally, sialylation of LNnT LOS occurs in organisms present in infected male urethral secretions [by electron microscopy (93)]. The importance of LNnT sialylation for virulence in humans was demonstrated in the experimental model of human infection that used a variant strain of *N. gonorrhoeae* that *in vitro* expressed predominantly Hep I linked lactose (Galβ1-4Glc; L8) but upon recovery from active infection, the sialylatable LNnT species predominated (65, 94). The terminal Gal of the P^*K*^-like structure from Hep I can also be sialylated (84) and recently, Hep II lactose has been shown to accept sialic acid (Figure 1) (78), which also inhibits complement deposition and engages Siglec (sialic acid-binding immunoglobulin-type lectin) receptors to down-regulate the host inflammatory response, thereby facilitating host immune evasion (95). Sialylation of the Hep II-attached lactose component of the 2C7 structure/epitope and sialylation (78) may contribute to gonococcal virulence provided by sialylation. Of note, mAb 2C7 continues to bind to *Ng* LOS even when the Hep I chain is extended beyond the minimal lactose structure (66), including binding to sialylated LNnT but less so when the P^*K*^ structure/epitope is expressed (96). Glycan extensions beyond lactose on Hep II, for example with GalNAc-Gal seen in a mutant strain selected under pyocin pressure called JW31R, abrogated mAb 2C7 binding (66). However, sialylation of Hep II lactose (78) variably affects binding of mAb 2C7 to gonococcal strains (78). Gonococcal strains that express the P^*K*^ structure/epitope are rare/absent *in vivo* (80, 97). Hep II extension beyond lactose, to our knowledge, has not been identified in strains isolated from humans, however, the recently identified additional acceptor site for sialic acid on Hep II lactose (78), suggests that strains bearing sialic acid at this site are likely to be present *in vivo*.

## The 2C7 Epitope and its Peptide Mimic

**Figure 3:**
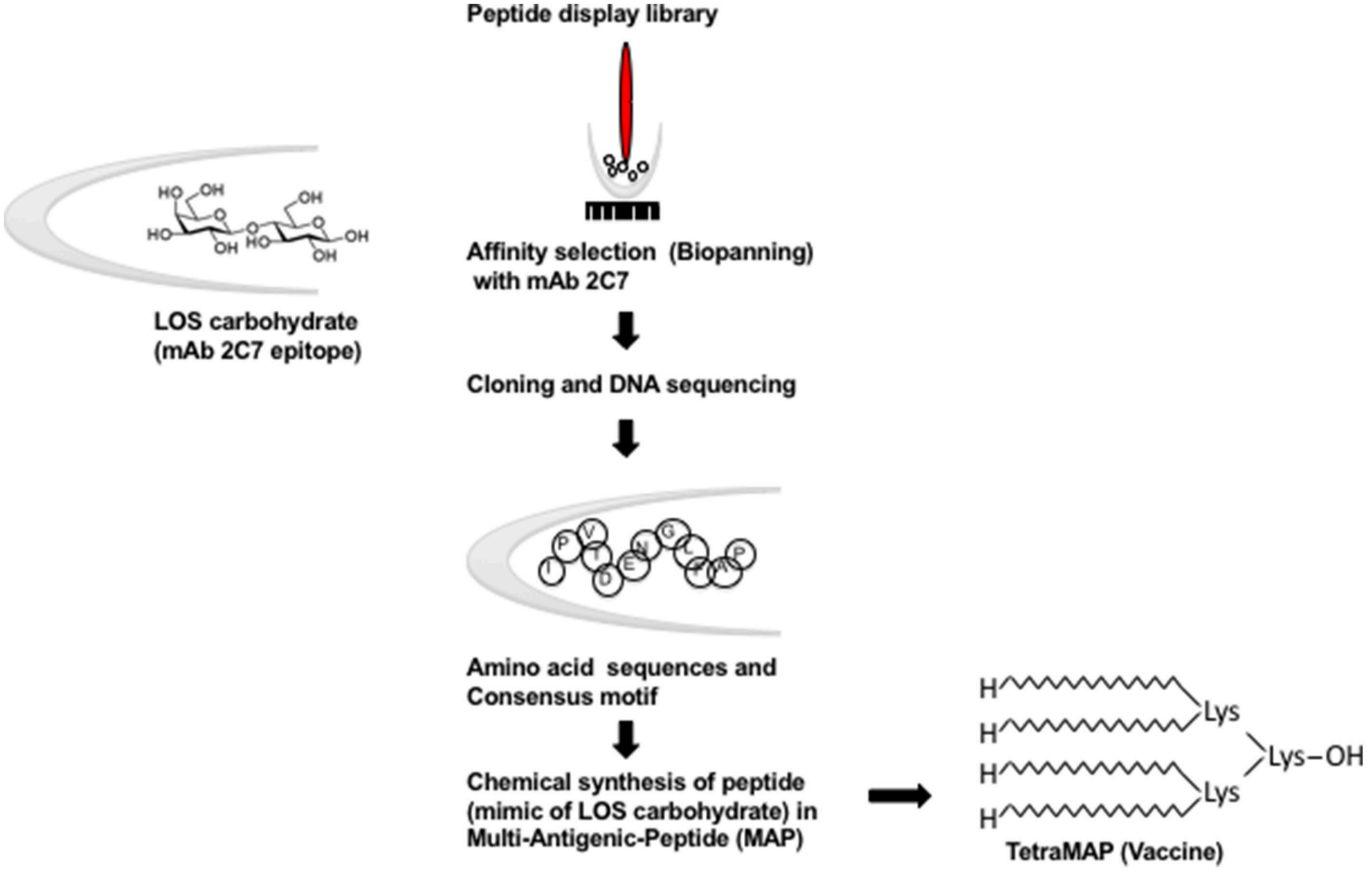
Schematic representation of the conversion of carbohydrate (OS) epitopes into peptide mimotopes. Peptide mimics of the 2C7 epitope were identified using a random FliTrx (Flagellin-Thioredoxin) peptide display library that were screened (Biopanned) using mAb 2C7 (98). The peptide–containing clones that bound to mAb 2C7 were eluted and sequenced. An optimal peptide that contained the consensus motif was synthesized as an octameric peptide (shown here as a multiantigenic peptide [MAP]) on a lysine backbone (TetraMAP), which is the currently used configuration.

The 2C7 OS epitope has been examined as a potential gonococcal candidate. Carbohydrate (OS) immunogens, themselves, evoke thymus-independent (TI) responses; they stimulate the production of low affinity IgM antibodies predominantly and there is no affinity maturation. Purification of OS from LOS may result in a change in configuration and thereby modify immunogenicity. Because the precise configurations of OS structures within intact LOSs are not known, synthesis would be difficult and optimizing the production of the correct isomers may not be possible without advance structural knowledge. The conversion of carbohydrate (OS) epitopes into peptide mimotopes having similar configuration (defined by recognition of the appropriate mAb, e.g., mAb 2C7 in the case of *N. gonorrhoeae*) is a means to overcome the TI nature of carbohydrate antigens (Figure 3). Peptide mimics of the 2C7 epitope were identified using a peptide display library that was screened using mAb 2C7 [a monoclonal Ab with complement-dependent bactericidal and opsonophagocytic activities (2)] and identified peptide mimics were down-selected immunochemically and for immunogenicity (98). Carbohydrates may contain multiple identical antigenic epitopes that provides a molecular configuration allowing carbohydrate to cross-link antigen to their cognate receptors on B cells. To emulate such configurations, an optimal peptide mimotope was chosen and a multiple antigen peptide (MAP) synthesized (Figure 3). Immunization of mice with peptide vaccine elicited cross-reactive anti-LOS antibodies that possessed dose responsive direct complement dependent bactericidal activity against gonococci (98). More recent refinements of the peptide building block have been directed: at stabilization to ensure homogeneity; optimization of synthesis to produce high yields and pairing of peptide vaccine with adjuvants that have been approved and used for human vaccination. Further characterization of vaccine induced immune responses evoked by the 2C7 peptide were enlisted to correlate efficacy of active vaccination with MAP in mice followed by experimental vaginal challenge with *Ng*. Mice immunized with MAP combined with monophosphoryl lipid A (MPL), a toll-like receptor 4 (TLR4) agonist, elicited a predominant complement-activating IgG subclass (IgG2a) response resulting from T_H_1-biased immune stimulation (7), similar to other vaccine strategies that have proved efficacious in the experimental murine model of *Ng* vaginal/cervical colonization (3, 5). Clearance of *Ng* infection was hastened in vaccinated mice and reduction of bacterial burdens occurred throughout the period of colonization (7). The level of vaccine induced 2C7 immune antibodies in the vaginas of mice correlated directly with reduction in bacterial burden (80). Results of active immunization with the peptide mimic were paralleled by similar results obtained with passive immunization of mAb 2C7 (7). These results strongly support a vaccine antibody-mediated effect that was dependent on the presence of local IgG antibody in mouse vaginas (80). 2C7 vaccine satisfies the three criteria proposed above for a gonococcal vaccine: (i) similar antigenic target representation across strains; (ii) a representative virulence determinant and (iii) a critical determinant for organism survival *in vivo*.

## Conclusion

Evidence that gonococcal vaccination can succeed in humans is encouraging. Although field trials with whole cell and pilus vaccines have been unsuccessful (99, 100), this occurred, in part, because of exposure of vaccine recipients to heterologous strains in the wild, different than were used to prepare vaccines. Homologous protection in human experimental infection was also shown to be possible in men with favorable antibody ratios directed against the strain used in experimental infection suggesting that protective immunity against broadly cross-reactive antigens will be necessary (15) while avoiding subversive effects that might otherwise undermine protective immune responses (101). Adaptation of such an antigen(s) could result in a successful vaccine. Recent epidemiologic evidence indicates that cross-reactivity between *N. meningitidis* and *N. gonorrhoeae* antigens induces a measurable level of cross protection (46), fulfilling, perhaps, the “triple threat” criteria indicated above that also applies to 2C7 vaccine: (i) broad representation; (ii) service as a virulence determinant and (iii) a critical role in organism survival.

## Author Contributions

SG and JS organized and prepared material for this manuscript. SR and PR contributed in the writing and reviewing of the manuscript.

### Conflict of Interest Statement

The authors declare that the research was conducted in the absence of any commercial or financial relationships that could be construed as a potential conflict of interest.
